# Emotional Awareness in Schizophrenia Is Associated With Gray Matter Volume of Right Precuneus

**DOI:** 10.3389/fpsyt.2021.601742

**Published:** 2021-04-01

**Authors:** Martin Jáni, Zora Kikinis, Jan Lošák, Ofer Pasternak, Filip Szczepankiewicz, Carina Heller, Sophia Swago, Annelise Silva, Sylvain Bouix, Marek Kubicki, Libor Ustohal, Petr Kudlička, Lubomír Vojtíšek, Carl-Frederik Westin, Tomáš Kašpárek

**Affiliations:** ^1^Department of Psychiatry, Faculty of Medicine, Masaryk University and University Hospital Brno, Brno, Czechia; ^2^Central European Institute of Technology, Masaryk University, Brno, Czechia; ^3^Department of Psychiatry, Brigham and Women's Hospital and Harvard Medical School, Boston, MA, United States; ^4^Department of Radiology, Brigham and Women's Hospital and Harvard Medical School, Boston, MA, United States; ^5^Medical Radiation Physics, Clinical Sciences, Lund University, Lund, Sweden; ^6^Department of Psychiatry and Psychotherapy, University Hospital Jena, Friedrich-Schiller-University Jena, Jena, Germany; ^7^VA Boston Healthcare System, Brockton Division, Brockton, MA, United States

**Keywords:** MRI, schizophrenia, psychopathology, emotional awareness, alexithymia

## Abstract

**Objectives:** We assessed the relationship between emotional awareness (e.g., the ability to identify and differentiate our own feelings and feelings of others) and regional brain volumes in healthy and in schizophrenia groups.

**Methods:** Magnetic resonance images of 29 subjects with schizophrenia and 33 matched healthy controls were acquired. Brain gray matter was parcellated using FreeSurfer and 28 regions of interest associated with emotional awareness were analyzed. All participants were assessed using the Levels of Emotional Awareness Scale (LEAS) of Self and of Other. LEAS scores were correlated with gray matter volume for each hemisphere on the 14 brain regions of the emotional awareness network.

**Results:** Individuals with schizophrenia showed decreased emotional awareness on both LEAS Self and LEAS Other compared to healthy controls. There were no statistically significant between-group differences in gray matter volumes of the emotional awareness network. The performance on LEAS Other correlated negatively with right precuneus gray matter volume only in the schizophrenia group.

**Conclusion:** Our findings suggest a relationship between gray matter volume of the right precuneus and deficits in understanding of emotional states of others in schizophrenia.

## Introduction

Schizophrenia is commonly associated with decreased everyday social functioning and quality of life. Previous research has focused primarily on neurocognition as a predictor of functional outcome. However, a meta-analysis of studies on neurocognition and on social cognition demonstrated that the strongest predictor of functional outcome in schizophrenia is social cognition (1). Though most social cognition research in schizophrenia has focused on theory of mind and the perception of emotions in others (2), emotional awareness of both, self and of others, has been reported to play an important role in functional outcome (3, 4).

Emotional awareness is the ability to consciously experience, describe and identify emotions (5). Deficits in emotional awareness have been observed in patients with schizophrenia and in other mental disorders. The term alexithymia, literally translated as a deficiency in reading emotions, was introduced to describe individuals with deficits in describing and identifying emotions, e.g., with diminished emotional awareness (6, 7). Emotional awareness is most often assessed by tests that are largely based on self-report, such as the Toronto Alexithymia Scale (TAS-20 and TAS-26) (8) or the Bermond-Vorst Alexithymia Questionnaire (BVAQ) (9). A meta-analysis (10) of clinical studies using the TAS-20 in schizophrenia reports that patients show difficulties in identifying and describing feelings, although the results were highly heterogeneous.

Emotional awareness is considered a cognitive ability developed during childhood and adolescence (11, 12) and its deficit can be regarded as a trait feature (13). It has not been established yet whether the deficits in emotional awareness in schizophrenia are associated with abnormalities in cortical gray matter. To our knowledge, only one study has investigated an association between gray matter volume and alexithymia measured by the self-report using the TAS-20 in schizophrenia (14). These investigators reported that decreased volume of left supramarginal gyrus was associated with higher alexithymia in subjects with schizophrenia but not in healthy controls.

In healthy populations, multiple brain regions have been proposed to be involved in emotional awareness. For example, a functional imaging study on healthy volunteers found direct engagement of the temporal pole, posterior and anterior cingulate cortex, medial frontal gyrus, inferior frontal gyrus, precuneus, right anterior insular cortex and ventromedial prefrontal cortex (VMPFC) when evaluating emotional states of self (15). In terms of neuroimaging studies of alexithymia, two meta-analyses in the general population have been conducted (see Table 1). First, Van der Velde et al. (16) focused on functional Magnetic Resonance Imaging (MRI) studies that measured brain activation while viewing emotional stimuli and the correlates of alexithymia were measured by either TAS-20 or BVAQ. They consistently found an association of alexithymia with increased activation of anterior cingulate cortex (ACC) while processing positive and negative emotional valence. In addition, alexithymia was associated with a diminished response of the amygdala, supplementary motor and premotor brain areas, and in the dorsomedial prefrontal cortex during the processing of negative stimuli. Decreased activation was also noted in the right insula and precuneus during processing of positive stimuli. Van der Velde et al. (16) speculated that decreased engagement of amygdala, right insula and precuneus is connected with decreased emotional awareness. A second meta-analysis by Xu and colleagues (17) focused on structural neuroimaging studies of alexithymia that used the self-report tests of TAS-20 or BVAQ. They reported reduced volume in the orbitofrontal and dorsolateral cortices, left insula, amygdala and striatal regions that is associated with increased alexithymia (17) (see Table 1). The above-mentioned studies are important in order to narrow down brain regions that might be involved in emotional awareness.

**Table 1:** Brain regions involved in emotional awareness across studies.

**Meta-analysis**	**Functional**	**Structural**
Region of interest	van der Velde et al. (16)	Xu et al. (17)
Lateral orbitofrontal		x
Medial orbitofrontal		x
Caudal middle frontal	x	
Rostral middle frontal	x	
Superior frontal	x	
Rostral anterior cingulate	x	
Caudal anterior cingulate	x	
Precuneus	x	
Cuneus	x	
Fusiform	x	x
Insula	x	
Amygdala	x	x
Caudate		x
Putamen		x

Notably, there are concerns regarding the assessment of emotional awareness using a self-report approach, as such assessments assume some level of insight and self-reflection that is difficult for some patients with schizophrenia. Thus, the use of self-report tests poses a risk of not detecting emotional awareness deficits (3). Therefore, in the current study, we use the Levels of Emotional Awareness Scale (LEAS), a written performance-based test, to control for the self-report problem. As part of the test, the participant is asked to describe their own feelings, and the feelings of another person in 20 different scenarios instead of just being asked in self-report “How good are you in describing feelings?” The score is based on the degree of differentiation in the use of emotion words, where more differentiated emotion words receive higher score, and represent greater awareness of emotional complexity (18). Lane et al. (19) suggests that the LEAS measures the person's ability to make mental representations of emotional states, while TAS-20 merely measures these difficulties in emotional awareness that are so extensive that the person is aware of his/her own deficit. Accordingly, studies using the LEAS have shown decreased emotional awareness in patients with schizophrenia (3, 20), even when the participant reported no self-perceived alexithymia (21).

Emotional awareness is closely related to theory of mind or perspective-taking, the ability to imagine and understand mental states of others (19). The problems with theory of mind constitutes one of the core features of schizophrenia (2). While the relationship of emotional awareness and theory of mind was also illustrated with LEAS overall score (19), there are some indications that the differentiation between emotional awareness for self and other would be beneficial. First, there is a possible conceptual distinction between the two, with emotional awareness for self-referring to the ability of identifying and describing one's own emotional states, while emotional awareness for others involves associated processes such as perspective-taking of others. Second, the benefit to differentiate for self and other is supported by studies where LEAS subscales for self and others were included and, accordingly, indicated distinct attributes of these subscales. Differences between schizophrenia and healthy controls were reported only in LEAS for self but not others (21). However, when only complex scenarios of LEAS were selected, schizophrenia patients had significantly lower score in LEAS for others (but not for self), which also solely correlated with the self-perceived quality of life (3). To summarize, the LEAS test is superior to the traditionally used self-report tests and the differentiation of assessing emotional awareness of self and other is an important feature to assess emotional awareness in people with schizophrenia and in healthy population.

In the present study, we investigated the relationship between brain structure and abnormalities in emotional awareness of self and others. We used the LEAS test to measure emotional awareness of self and others, in healthy controls and in patients with schizophrenia. We used structural MRI of the brain to determine volumes of gray matter regions that were identified as relevant for emotional awareness in previously published studies (see Table 1 for list of regions). To uncover the association between brain regions and emotional awareness, we correlated the scores on LEAS Self and LEAS Other with the volumes of the brain regions. We hypothesized that reduced volumes of gray matter regions would be associated with lower performance on emotional awareness.

## Methods

### Participants

The study included 29 (11 female) schizophrenia subjects and 33 (13 female) age, education and gender matched control subjects. The patients were in stable condition, recruited while they were either inpatients (hospitalized for a psychotic episode, during their last week at the hospital) or outpatients in the Department of Psychiatry at University Hospital Brno, Brno, Czech Republic. The diagnosis of schizophrenia was assessed using the Structured Clinical Interview for DSM5–Research version (SCID-RV) criteria (22). Schizophrenia symptoms were evaluated using the Positive and Negative Symptoms Scale (PANSS) (23). Those diagnosed with schizophrenia were taking antipsychotic medication. The healthy controls were recruited using advertising within the local community, Brno, Czech Republic and had no history of psychiatric illness themselves [assessed with Mini-International Neuropsychiatric Interview (24)] or up to second degree relatives. Exclusion criteria for both groups included history of neurological injury, brain disorder, substance abuse, and inability to undergo MRI. All participants signed informed consent. The study was approved by the Institutional Ethical committee of the University Hospital Brno.

### Procedures

#### Emotional Awareness

The Levels of Emotional Awareness Scale, LEAS (18) consists of 20 written descriptions of situations designed to elicit four emotions: anger, fear, happiness, or sadness. After reading each situation, the participant responded in writing to two questions: “*How would you feel?”* and “*How would the other person feel?*” This resulted in two subscales: first, emotional awareness for self (LEAS Self) and second, emotional awareness for others (LEAS Other). Qualitative assessment of responses is described in detail in the manual. In short, the test quantifies the participant's ability to describe specific emotions. The more elaborate descriptions of feelings are scored higher on a scale between 0 to 4, that is a score of 0 for reflection of cognitive states, score of 1 for bodily or physical sensations, score of 2 for undifferentiated feelings like “good” or “bad,” score of 3 for specific elaborate emotions like “sad” or “disappointed” and score of 4 for combination of two or more elaborate and differentiated emotions. In total, higher score indicates higher levels of emotional awareness. The LEAS consists of two versions, A and B, each consisting of 10 situations. Version A of LEAS was used in this study. LEAS has been validated as a reliable tool for measurement of alexithymia (25).

#### General Cognition

Neurocognition was assessed using the Czech version (26) of the MATRICS Consensus Cognitive Battery [MCCB, (27)]. MCCB composite score was computed without the managing emotions subtest. It has been recommended recently to exclude this emotional subtest from MCCB and report it separately as a domain of social cognition (28).

### MRI Acquisition and Image Postprocessing

The structural MRI data were collected on a 3T Siemens Magnetom Prisma (Erlangen, Germany) with 64 channel Head-Neck coil. Subjects lay in the supine position with their heads supported and immobilized using foam padding. A whole brain, high-resolution three-dimensional T1-weighted magnetization prepared rapid gradient echo (MPRAGE) sequence scan was collected (240 sagittal slices, field of view = 224 × 224 mm^2^, 1 mm^3^ isotropic voxel, TR = 2.3 s, TE = 2.33 ms, flip angle = 8°).

Volumetric measures of brain regions were obtained from MRI data using FreeSurfer, Version 6 (http://surfer.nmr.mgh.harvard.edu). FreeSurfer software performs a surface-based estimation of regional gray matter volume and parcellates each individual brain in an automated approach into 78 cortical and seven subcortical regions (29). Based on the published meta-analyses of neuroimaging studies of alexithymia (16, 17), we focused on brain regions that were relevant for emotional awareness (Table 1) and selected the corresponding 28 regions of interest (ROI), 14 in each hemisphere, from FreeSurfer parcellations. We normalized the volumes using total brain volume excluding ventricles to control for the size of the head.

### Statistical Analysis

First, we assessed the normality of our data. Volumes of nine gray matter regions were not distributed normally (medial orbitofrontal cortex, precuneus, insula, caudate and putamen in the left hemisphere and caudal middle frontal gyrus, precuneus, cuneus and caudate in the right hemisphere). To achieve normal distribution of the data, all volumes were transformed with log-10 transformation. The log transformed data was used in further analyses.

Between group differences (schizophrenia patients and healthy controls) were analyzed using ANCOVA, with age and sex included as covariates of no interest. Then, we evaluated the interaction effect of group and gray matter volume on emotional awareness. We used hierarchical model with first controlling for age and sex, then adding main effects of group and ROI volume and finally the interaction effect. Separate models were used for each ROI and LEAS subscale. Subsequently, the association between volumes of the brain regions and emotional awareness was assessed in each group separately by Spearman's ρ to counter for possible non-linear relationship. For the healthy control group, we conducted partial correlations with age, sex and MCCB composite score as covariates. For the schizophrenia group, in addition to age, sex and MCCB, to counter the clinical confounds, we also included medication, PANSS total score and number of episodes as covariates. Finally, we conducted additional bivariate correlations to assess relationship between LEAS subscales and demographic/clinical variables. As the normalization of the Czech version of MCCB is still in progress, we used standardized z-scores for neurocognition. We used False-discovery rate (FDR) to correct for multiple testing of 28 selected ROIs (14 ROIs on each of the brain hemisphere). Alpha level was set to 0.05 for all tests with FDR correction. Statistic tests were computed using JASP (JASP Team, version 0.9), SPSS (IBM Corp., version 23.0) and R (R Core Team, version 3.6.0).

## Results

### Demographics and Neurocognition

The groups did not differ in age, gender nor education. Patients showed statistically significant cognitive deficit in all MCCB domains (Table 2).

**Table 2:** Demographical, clinical and neurocognitive characteristics.

		**SZ (*****n*** **=** **29)**				**HC (*****n*** **=** **33)**						
		**Min**	**Max**	**Mean**	**SD**	**Min**	**Max**	**Mean**	**SD**	***t***	**df**	***p***
**Demographic**												
	Age (years)	18	49	32.45	9.01	17	55	32.3	9.17	−0.06	60	0.950
	Education (years)	9	20	13.93	2.85	9	18	14.82	2.70	1.26	60	0.213
	Gender	18 male/11 female				20 male/13 female				χ^2^ = 0.16	1	0.899
	Handedness	one left/28 right				33 right				χ^2^ = 1.16	1	0.282
**Clinical**												
	Illness duration (years)	0	22	7.69	6.48							
	Number of episodes	1	10	3.46	2.43							
	Medication (CPZ mg/day)	150	1.750	724.93	381.82							
	PANSS Total	30	92	56.45	15.18							
	PANSS Positive	7	25	11.72	4.84							
	PANSS Negative	7	29	15.34	6.26							
	PANSS General	16	48	29.38	7.24							
**MCCB (*****Z*****-scores)**												
	Speed of processing	−2.95	0.68	−0.70	0.90	−0.55	1.8	0.62	0.60	6.86	60	<0.001
	Working memory	−2.54	0.77	−0.64	0.87	−0.71	2.43	0.56	0.74	5.88	60	<0.001
	Verbal learning	−2.86	1.05	−0.58	1.06	−0.9	1.64	0.51	0.59	4.89	42.45	<0.001
	Visual learning	−2.80	0.89	−0.46	1.03	−2.21	1.33	0.40	0.78	3.68	51.98	0.001
	Planning/Decision-making	−2.27	1.02	−0.47	1.09	−1.98	1.02	0.41	0.70	3.75	46.48	<0.001
	Attention/vigilance	−3.28	1.16	−0.68	0.88	−0.53	2.01	0.59	0.67	6.43	60	<0.001
	Neurocognitive composite	−2.93	0.68	−0.75	0.89	−0.41	1.42	0.66	0.49	7.56	42.3	<0.001

*SZ. schizophrenia; HC. healthy controls; SD. standard deviation; df. degrees of freedom; CPZ. chlorpromazine equivalents; PANSS. Positive and negative syndromes scale; MCCB. MATRICS consensus cognitive scale*.

### Group Differences in Emotional Awareness

Most notably, patients presented with statistically significant decreases in total scores of emotional awareness, on both LEAS subscales, the emotional awareness for self and for others (Table 3).

**Table 3:** Group differences in emotional awareness.

	**SZ (*****n*** **=** **29)**				**HC (*****n*** **=** **33)**						
	**Min**	**Max**	**Mean**	**SD**	**Min**	**Max**	**Mean**	**SD**	***t***	**df**	***p***
LEAS total	14	39	26.72	6.35	23	38	30.64	4.31	2.87	60.00	0.006
LEAS Self	12	33	23.38	5.43	12	36	27.24	5.24	2.85	60.00	0.006
LEAS Other	5	34	21.07	7.63	16	32	24.76	4.55	2.27	44.43	0.028

### Group Differences in Brain Region Volumes

ROI analysis revealed no statistically significant differences after FDR correction (see Supplementary Table 1).

### The Interaction of Group by Brain Region Volumes Effect on Emotional Awareness

We found significant effect on emotional awareness by interaction of group with right precuneus volume accounting for 38.1% of variation [and 15.4% of unique variation, *F*_(6, 54)_ = 5.54, *p* = 0.016, FDR corrected], when controlling for age and sex [accounting for 18% of variation, *F*_(3, 57)_ = 4.21, *p* = 0.009] and including main effects of group and gray matter volume of the ROI [4.5% of variation, *F*_(2, 55)_ = 1.61, *p* = 0.208]. No other interaction effect was significant. Complete statistics for model of each ROI are given in the Supplementary Table 2.

### Correlations Between Brain Regions Volumes and Emotional Awareness

**Figure 1:**
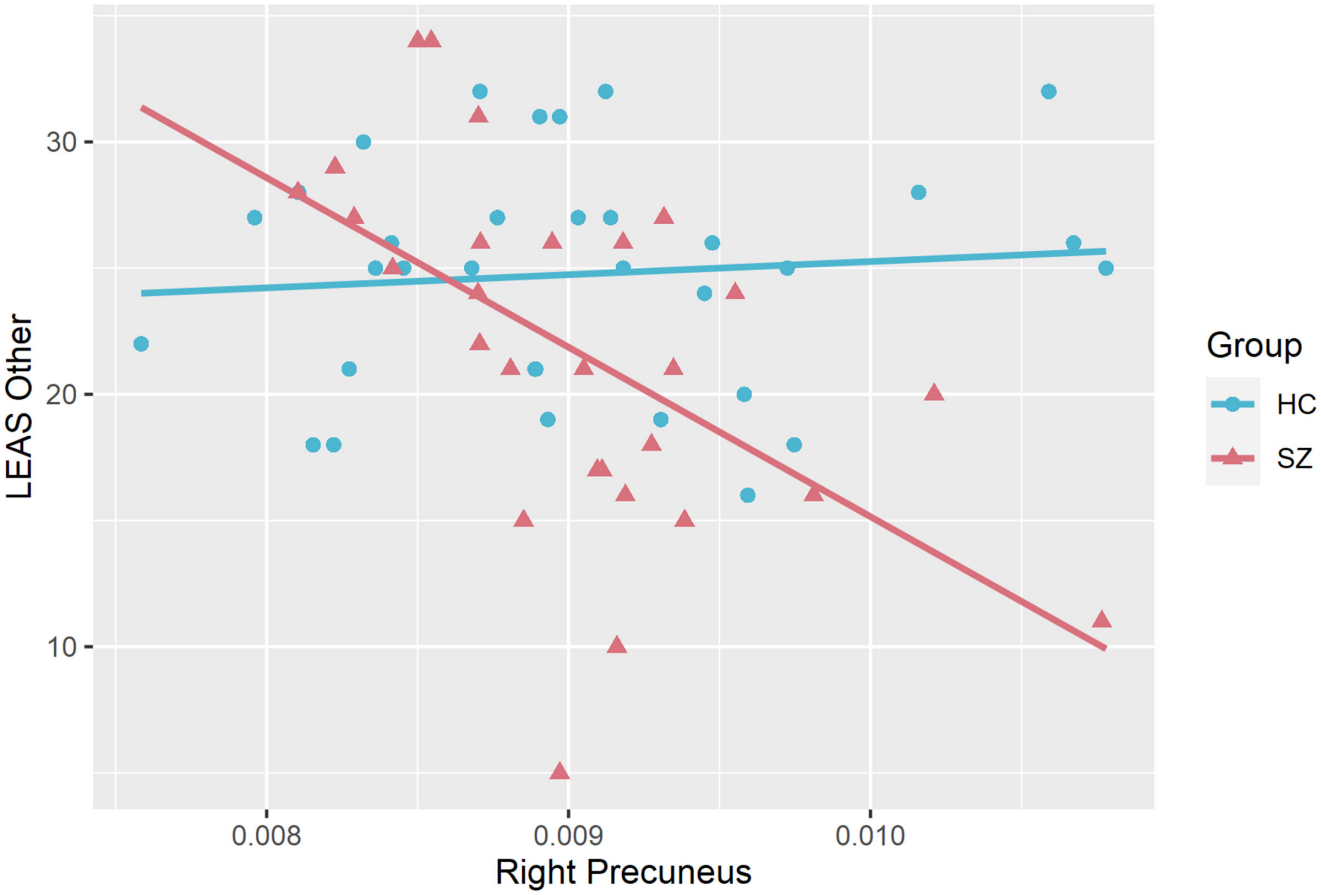
Correlations between emotional awareness for others (LEAS Other) and gray matter volume of right precuneus significant in schizophrenia group (SZ) but not in healthy controls (HC).

The scores on LEAS Other were highly significantly and negatively associated with the volume of right precuneus in schizophrenia group (*r* = −0.68, *p* < 0.05, FDR corrected, controlled for age, sex, medication, MCCB, PANSS and number of episodes). This relationship was non-significant in the control group. The relationship between LEAS Other and the volume of right precuneus in both groups is plotted in Figure 1. The results of correlations of all brain regions are given in the Supplementary Table 3.

### Additional Analyses

We explored whether demographic, clinical or general cognition functioning were related to emotional awareness. No significant correlations between psychopathology (PANSS), antipsychotic medication, illness duration, or neurocognition (MCCB composite) with emotional awareness were found. We found significant correlations between age and LEAS Self in the healthy group (*r* = −0.48, *p* = 0.005) and in the combined group (*r* = −0.36, *p* = 0.004). We also found significant correlations between verbal learning and LEAS Self in the healthy group (*r* = 0.56, *p* = 0.005) and in the combined group (*r* = 0.57, *p* < 0.001). No differences were found between gender and in LEAS Self (*p* = 0.108) or LEAS Other (*p* = 0.093).

## Discussion

On the behavioral level, we found decreased emotional awareness for both LEAS Self and LEAS Other in the schizophrenia group when compared to the healthy controls, which is in accordance with previously published findings (20). Although some studies suggest selective impairment of emotional awareness of either only self (21), or others (3), our study supports a general impairment of emotional awareness in individuals with schizophrenia. We did not find any association between LEAS and PANSS subscales, illness duration, medication or neurocognition (MCCB composite). To the best of our knowledge, the association between anatomical brain substrates and the LEAS is a novel finding. Increased score on LEAS Other was associated with decreased volume of the right precuneus in patients with schizophrenia, but not in the healthy control group.

Findings of no volumetric differences between schizophrenia and healthy controls is surprising, but not unexpected. Meta-analytic findings report widespread frontotemporal and subcortical reductions of gray matter volume in schizophrenia (30, 31). Our negative findings might be attributed to the relatively small sample and subtle differences that might have remained undetected, while volumetric studies usually require large samples to find between subject differences. In addition, the large variance of age in our sample (including both first episodes and chronic patients) might have obscured the results, as gray matter volume alterations in schizophrenia are characterized by progressive change during the lifespan (32, 33).

### Relationship Between Gray Matter Volume in Right Precuneus and Emotional Awareness for Others

Performance on LEAS Other was associated with right precuneus volume. Interestingly, this association was present in the schizophrenia group but not in healthy subjects, suggesting an association specific to schizophrenia between this region and emotional awareness for others. In contrast to our hypothesis of smaller volumes associated with lower performance on emotional awareness, we found that larger volume of right precuneus is associated with LEAS for others. This association was significant even when controlling for age, sex, neurocognition and clinical (psychopathology symptoms, number of episodes and medication) effects.

Precuneus plays major role in higher order self-processes, including shifting between 1st and 3rd person perspective (34) and the attribution of emotion to self and others (35). Previously published research shows functional abnormalities in precuneus in connection to schizophrenia, such as that patients with schizophrenia show altered activity in precuneus when taking the perspective of others (36). In our study while we do not find any changes in volume of the precuneus between the healthy and the schizophrenia group, we report that increased precuneus volume was associated with decreased emotional awareness to others in the schizophrenia group. In the general population, the precuneus is involved in perspective-taking by assigning first-person perspective (35, 37, 38), and the increase of its gray matter volume is associated with greater tendency to recall episodic memories from egocentric perspective (39, 40). Therefore, we speculate that increased precuneus volume might contribute to *egocentricity bias*, that is a failure to suppress one's own perspective in order to be able to effectively imagine the perspective of others (41). The larger precuneus volume in schizophrenia group might then lead to exuberant focus on egocentric perspective, thus failing to switch the perspective from self to the others when asked about emotional states of others. This might in turn lead to decreased ability to attribute emotions effectively and impair the emotional awareness for others.

Apart from right precuneus, we found no relationship between gray matter volume and emotional awareness in schizophrenia nor in healthy controls. Previous studies indicate association between alexithymia and structural brain changes in general population (17) and also in schizophrenia (14). First, it is possible that our healthy controls did not show enough variance to detect any impairment in emotional awareness. Second, our study is novel in that it used performance-based assessment of emotional awareness, the LEAS, while previous studies relied on self-report questionnaires, limiting the comparability. In addition, given the nature of the emotional awareness, self-report measures can only detect deficit that is so severe that is apparent also to the participant (19), while LEAS might detect subtle deficits, that are not evident in the gray matter alterations.

### Limitations

There are several limitations concerning this study. First, this is an exploratory correlation study, investigating the associations between subject's performance on LEAS and the volumes of specific brain regions. Because we selected 14 specific brain regions that were reported earlier as the areas associated with emotional awareness, we did not explore the entire brain and thus our results are limited to those regions only. In addition, we explored only the volume, the most studied measure in emotional awareness, providing us a framework for our research, a hypothesis and comparability with previous studies. Measures of cortical thickness and cortical areas should be included in future studies. Second, we used FreeSurfer to parcellate the brain into anatomical areas, although volumetric changes in smaller, but functionally relevant subareas might remain undetected. Third, several variables could have influenced the results. Clinical variables such as state of psychosis, antipsychotic medication, or illness duration did not correlate with LEAS, which is in line with previous studies using LEAS in schizophrenia (3, 20, 21). Therefore, the effect of clinical variables is unlikely, suggesting rather stable, trait like nature of the emotional awareness deficit. However, the emotional awareness of self deteriorates with age, which is in accordance with previous research (42). This age effect was observed in the combined group and in the healthy control group, but not in the schizophrenia group, which could possibly be explained by abnormal neurodevelopmental trajectory of the general cognitive impairment in schizophrenia, which has been established even before the prodromal phase and remains stable during remission (43).

## Conclusion

We report that patients with schizophrenia exhibit less emotional awareness on both, the LEAS Self and LEAS Other, subtests. We also report that scores on the LEAS Other correlated with one specific region, the right precuneus, in the schizophrenia group, but not in the healthy group. In conclusion, we demonstrated that dysfunction in understanding of emotional states of others is related to a distinct neurobiological substrate, and that volumetric alterations of the right precuneus constitute a key pathology underlying the reduced emotional awareness of others observed in schizophrenia.

## Data Availability Statement

The raw data supporting the conclusions of this article will be made available by the authors, without undue reservation.

## Ethics Statement

The studies involving human participants were reviewed and approved by University Etická komise FN Brno (Ethics committee of University Hospital Brno) Jihlavská 20, 625 00 Brno, Czech Republic. The patients/participants provided their written informed consent to participate in this study.

## Author Contributions

MJ: conceptualization, methodology, formal analysis, investigation, writing - original draft, visualization, and data curation. ZK: conceptualization, methodology, validation, resources, formal analysis, writing - original draft, and data curation. JL: investigation, writing - review and editing, and data curation. OP, FS, CH, SS, AS, and SB: software, validation, writing - review, and editing. MK: resources, writing - review and editing, and funding acquisition. LU: investigation, writing - review, and editing. PK: software, validation, investigation, resources, writing - review, and editing. LV: software, validation, resources, writing - review, and editing. C-FW: software, validation, resources, writing - review and editing, and funding acquisition. TK: conceptualization, writing - review and editing, supervision, project administration, funding acquisition, and resources. All authors contributed to the article and approved the submitted version.

## Conflict of Interest

The authors declare that the research was conducted in the absence of any commercial or financial relationships that could be construed as a potential conflict of interest.
