# Supplementary material for: Emotional Awareness in Schizophrenia Is Associated With Gray Matter Volume of Right Precuneus
Source: Front Psychiatry. 2021 Apr 1;12:601742. doi: 10.3389/fpsyt.2021.601742 (PMC8046932; doi:10.3389/fpsyt.2021.601742)
Supplement: Supplementary file 1 [file Data_Sheet_1.PDF]

Supplementary Material

Supplementary table 1. Between group comparison of gray matter volumes in selected regions of interest with age and sex as covariates.

					Healthy controls			Schizophrenia		
**Region of interest**	**F**	**df**	**p**	**Partial Eta Squared**	**Mean**	**SD**	**SE**	**Mean**	**SD**	**SE**
left lateral orbitofrontal	0.085	1, 59	0.93	<0.01	0.002	4.351e -4	7.462e -5	0.002	3.493e -4	6.376e -5
left medial orbitofrontal	0.187	1, 59	0.88	<0.01	0.004	4.672e -4	8.012e -5	0.004	3.556e -4	6.492e -5
left caudal middle frontal	0.062	1, 59	0.93	<0.01	0.005	7.659e -4	1.313e -4	0.006	6.061e -4	1.107e -4
left rostral middle frontal	1.203	1, 59	0.62	0.02	0.014	0.002	2.726e -4	0.014	0.001	2.511e -4
left superior frontal	2.657	1, 59	0.36	0.04	0.019	0.002	2.898e -4	0.019	0.002	3.776e -4
left rostral anterior cingulate	5.044	1, 59	0.28	0.08	0.002	3.401e -4	5.832e -5	0.002	3.189e -4	5.822e -5
left caudal anterior cingulate	1.200	1, 59	0.62	0.02	0.002	3.552e -4	6.092e -5	0.002	3.965e -4	7.239e -5
left precuneus	0.476	1, 59	0.82	0.01	0.009	7.730e -4	1.326e -4	0.009	6.047e -4	1.104e -4
left cuneus	2.876	1, 59	0.36	0.05	0.003	3.624e -4	6.215e -5	0.003	4.515e -4	8.243e -5
left fusiform	0.124	1, 59	0.91	<0.01	0.009	7.270e -4	1.247e -4	0.009	0.001	1.855e -4
left insula	0.436	1, 59	0.82	0.01	0.006	5.233e -4	8.974e -5	0.006	5.723e -4	1.045e -4
left amygdala	4.144	1, 59	0.34	0.07	0.002	1.963e -4	3.367e -5	0.001	1.633e -4	2.982e -5
left caudate	0.810	1, 59	0.75	0.01	0.004	4.984e -4	8.547e -5	0.004	5.015e -4	9.157e -5
left putamen	3.085	1, 59	0.36	0.05	0.005	6.761e -4	1.160e -4	0.005	5.509e -4	1.006e -4
right lateral orbitofrontal	0.010	1, 59	1.00	<0.01	0.006	4.641e -4	7.959e -5	0.006	7.710e -4	1.408e -4
right medial orbitofrontal	0.323	1, 59	0.87	0.01	0.005	4.194e -4	7.193e -5	0.005	4.378e -4	7.992e -5
right caudal middle frontal	0.752	1, 59	0.75	0.01	0.005	8.772e -4	1.504e -4	0.005	6.500e -4	1.187e -4
right rostral middle frontal	0.001	1, 59	1.00	<0.01	0.014	0.002	3.634e -4	0.014	0.002	2.940e -4
right superior frontal	6.551	1, 59	0.19	0.10	0.019	0.001	2.416e -4	0.018	0.001	2.489e -4
right rostral anterior cingulate	0.622	1, 59	0.79	0.01	0.002	2.877e -4	4.934e -5	0.002	2.792e -4	5.097e -5
right caudal anterior cingulate	3.721	1, 59	0.34	0.06	0.002	3.353e -4	5.751e -5	0.002	3.483e -4	6.360e -5
right precuneus	<0.001	1, 59	1.00	<0.01	0.009	7.729e -4	1.326e -4	0.009	7.322e -4	1.337e -4
right cuneus	1.555	1, 59	0.57	0.03	0.003	4.420e -4	7.580e -5	0.003	4.271e -4	7.797e -5
right fusiform	2.439	1, 59	0.36	0.04	0.009	8.975e -4	1.539e -4	0.008	8.782e -4	1.603e -4
right insula	0.192	1, 59	0.88	<0.01	0.006	5.714e -4	9.800e -5	0.006	5.763e -4	1.052e -4
right amygdala	<0.001	1, 59	1.00	<0.01	0.002	1.473e -4	2.526e -5	0.002	1.928e -4	3.519e -5
right caudate	0.204	1, 59	0.88	<0.01	0.004	5.166e -4	8.859e -5	0.004	4.948e -4	9.035e -5
right putamen	2.477	1, 59	0.36	0.04	0.005	6.153e -4	1.055e -4	0.005	5.533e -4	1.010e -4

Supplementary table 2a. Hierarchical model of gray matter volume by group interaction with LEAS Self score.

					Change Statistics					full model p
**Region of interest**	**Model**	**R**	**R²**	**SE**	**R²**	**F**	**df1**	**df2**	**p**	**(FDR corr.)**
(covariates)	age & sex	0,506	0,256	4,781	0,256	6,550	3	57	0,001	
left lateral orbitofrontal	& main effects	0,585	0,342	4,579	0,085	3,565	2	55	0,035	
	& interaction	0,610	0,372	4,512	0,031	2,644	1	54	0,110	0,512
left medial orbitofrontal	& main effects	0,654	0,428	4,268	0,172	8,251	2	55	0,001	
	& interaction	0,654	0,428	4,307	0,000	0,020	1	54	0,889	0,964
left caudal middle frontal	& main effects	0,573	0,329	4,624	0,072	2,966	2	55	0,060	
	& interaction	0,585	0,343	4,618	0,014	1,131	1	54	0,292	0,682
left rostral middle frontal	& main effects	0,563	0,317	4,665	0,060	2,425	2	55	0,098	
	& interaction	0,563	0,317	4,708	0,000	0,008	1	54	0,930	0,964
left superior frontal	& main effects	0,595	0,354	4,536	0,098	4,161	2	55	0,021	
	& interaction	0,596	0,355	4,575	0,001	0,050	1	54	0,823	0,964
left rostral anterior cingulate	& main effects	0,590	0,348	4,558	0,091	3,848	2	55	0,027	
	& interaction	0,590	0,348	4,600	0,000	0,000	1	54	0,990	0,990
left caudal anterior cingulate	& main effects	0,563	0,317	4,666	0,060	2,423	2	55	0,098	
	& interaction	0,569	0,324	4,685	0,007	0,554	1	54	0,460	0,805
left precuneus	& main effects	0,563	0,317	4,666	0,060	2,421	2	55	0,098	
	& interaction	0,609	0,371	4,519	0,054	4,636	1	54	0,036	0,512
left cuneus	& main effects	0,580	0,336	4,597	0,080	3,321	2	55	0,043	
	& interaction	0,588	0,346	4,607	0,009	0,774	1	54	0,383	0,715
left fusiform	& main effects	0,569	0,324	4,639	0,068	2,763	2	55	0,072	
	& interaction	0,589	0,347	4,604	0,022	1,843	1	54	0,180	0,570
left insula	& main effects	0,563	0,317	4,666	0,060	2,423	2	55	0,098	
	& interaction	0,606	0,367	4,532	0,050	4,282	1	54	0,043	0,512
left amygdala	& main effects	0,569	0,324	4,641	0,067	2,735	2	55	0,074	
	& interaction	0,569	0,324	4,683	0,000	0,035	1	54	0,853	0,964
left caudate	& main effects	0,568	0,322	4,646	0,066	2,672	2	55	0,078	
	& interaction	0,602	0,362	4,550	0,040	3,354	1	54	0,073	0,512
left putamen	& main effects	0,566	0,320	4,654	0,064	2,570	2	55	0,086	
	& interaction	0,593	0,352	4,586	0,032	2,645	1	54	0,110	0,512
right lateral orbitofrontal	& main effects	0,566	0,321	4,652	0,064	2,604	2	55	0,083	
	& interaction	0,588	0,346	4,606	0,025	2,103	1	54	0,153	0,570
right medial orbitofrontal	& main effects	0,563	0,317	4,664	0,061	2,444	2	55	0,096	
	& interaction	0,565	0,319	4,701	0,002	0,133	1	54	0,716	0,964
right caudal middle frontal	& main effects	0,563	0,317	4,666	0,060	2,421	2	55	0,098	
	& interaction	0,579	0,336	4,642	0,019	1,554	1	54	0,218	0,610
right rostral middle frontal	& main effects	0,566	0,320	4,654	0,064	2,568	2	55	0,086	
	& interaction	0,566	0,320	4,696	0,000	0,033	1	54	0,857	0,964
right superior frontal	& main effects	0,615	0,378	4,452	0,121	5,361	2	55	0,007	
	& interaction	0,623	0,388	4,457	0,010	0,885	1	54	0,351	0,702
right rostral anterior cingulate	& main effects	0,565	0,319	4,656	0,063	2,548	2	55	0,087	
	& interaction	0,569	0,324	4,682	0,005	0,381	1	54	0,540	0,840
right caudal anterior cingulate	& main effects	0,563	0,317	4,666	0,060	2,421	2	55	0,098	
	& interaction	0,568	0,322	4,689	0,006	0,445	1	54	0,508	0,836
right precuneus	& main effects	0,563	0,317	4,666	0,060	2,423	2	55	0,098	
	& interaction	0,582	0,339	4,631	0,022	1,817	1	54	0,183	0,570
right cuneus	& main effects	0,583	0,340	4,584	0,084	3,502	2	55	0,037	
	& interaction	0,584	0,341	4,623	0,001	0,062	1	54	0,805	0,964
right fusiform	& main effects	0,583	0,340	4,584	0,084	3,491	2	55	0,037	
	& interaction	0,584	0,341	4,624	0,001	0,058	1	54	0,810	0,964
right insula	& main effects	0,573	0,329	4,623	0,072	2,970	2	55	0,060	
	& interaction	0,586	0,343	4,617	0,014	1,160	1	54	0,286	0,682
right amygdala	& main effects	0,569	0,323	4,643	0,067	2,719	2	55	0,075	
	& interaction	0,569	0,323	4,685	0,000	0,011	1	54	0,917	0,964
right caudate	& main effects	0,563	0,317	4,664	0,061	2,442	2	55	0,096	
	& interaction	0,573	0,328	4,669	0,011	0,890	1	54	0,350	0,702
right putamen	& main effects	0,563	0,317	4,666	0,060	2,422	2	55	0,098	
	& interaction	0,590	0,349	4,597	0,032	2,659	1	54	0,109	0,512

Hierarchical model with 3 steps: 1st step including covariates of age and sex; 2nd step including main effects of group and Region of interest; 3rd step including interaction between main effects. Abbreviations: LEAS = Levels of emotional awareness scale, SE = standard error, df = degrees of freedom, FDR = false discovery rate.

Supplementary table 2b. Hierarchical model of gray matter volume by group interaction with LEAS Other score.

Region of interest	Model	R	R^2	SE	Change Statistics R^2	F	df1	df2	p	full model p (FDR corr.)
(covariates)	age & sex	0,426	0,181	5,584	0,181	4,212	3	57	0,009	
left lateral orbitofrontal	& main effects	0,462	0,214	5,572	0,032	1,129	2	55	0,331	
	& interaction	0,490	0,240	5,530	0,026	1,839	1	54	0,181	0,562
left medial orbitofrontal	& main effects	0,500	0,250	5,441	0,069	2,522	2	55	0,090	
	& interaction	0,506	0,256	5,471	0,006	0,402	1	54	0,529	0,871
left caudal middle frontal	& main effects	0,450	0,202	5,612	0,021	0,717	2	55	0,493	
	& interaction	0,499	0,249	5,496	0,046	3,341	1	54	0,073	0,353
left rostral middle frontal	& main effects	0,439	0,193	5,646	0,011	0,383	2	55	0,683	
	& interaction	0,444	0,197	5,682	0,005	0,307	1	54	0,582	0,880
left superior frontal	& main effects	0,440	0,194	5,642	0,012	0,418	2	55	0,660	
	& interaction	0,455	0,207	5,647	0,013	0,907	1	54	0,345	0,758
left rostral anterior cingulate	& main effects	0,478	0,228	5,520	0,047	1,667	2	55	0,198	
	& interaction	0,478	0,228	5,570	0,000	0,015	1	54	0,904	0,904
left caudal anterior cingulate	& main effects	0,438	0,192	5,647	0,011	0,369	2	55	0,693	
	& interaction	0,440	0,193	5,695	0,001	0,082	1	54	0,776	0,894
left precuneus	& main effects	0,461	0,213	5,574	0,031	1,100	2	55	0,340	
	& interaction	0,504	0,255	5,475	0,042	3,012	1	54	0,088	0,353
left cuneus	& main effects	0,467	0,218	5,557	0,037	1,285	2	55	0,285	
	& interaction	0,481	0,231	5,560	0,013	0,937	1	54	0,337	0,758
left fusiform	& main effects	0,440	0,194	5,642	0,012	0,417	2	55	0,661	
	& interaction	0,476	0,227	5,576	0,033	2,320	1	54	0,134	0,468
left insula	& main effects	0,439	0,193	5,646	0,011	0,379	2	55	0,686	
	& interaction	0,526	0,277	5,394	0,084	6,267	1	54	0,015	0,215
left amygdala	& main effects	0,438	0,192	5,647	0,011	0,368	2	55	0,694	
	& interaction	0,456	0,208	5,643	0,016	1,082	1	54	0,303	0,758
left caudate	& main effects	0,438	0,192	5,647	0,011	0,368	2	55	0,694	
	& interaction	0,447	0,200	5,671	0,008	0,538	1	54	0,466	0,820
left putamen	& main effects	0,459	0,211	5,583	0,029	1,015	2	55	0,369	
	& interaction	0,460	0,212	5,629	0,001	0,096	1	54	0,757	0,894
right lateral orbitofrontal	& main effects	0,439	0,193	5,645	0,011	0,392	2	55	0,678	
	& interaction	0,448	0,201	5,669	0,008	0,533	1	54	0,468	0,820
right medial orbitofrontal	& main effects	0,442	0,196	5,635	0,014	0,488	2	55	0,617	
	& interaction	0,499	0,249	5,496	0,053	3,823	1	54	0,056	0,353
right caudal middle frontal	& main effects	0,458	0,210	5,584	0,029	1,000	2	55	0,374	
	& interaction	0,505	0,255	5,474	0,045	3,227	1	54	0,078	0,353
right rostral middle frontal	& main effects	0,445	0,198	5,626	0,017	0,578	2	55	0,565	
	& interaction	0,446	0,199	5,675	0,001	0,047	1	54	0,830	0,894
right superior frontal	& main effects	0,439	0,192	5,647	0,011	0,374	2	55	0,690	
	& interaction	0,453	0,205	5,653	0,013	0,882	1	54	0,352	0,758
right rostral anterior cingulate	& main effects	0,439	0,192	5,647	0,011	0,370	2	55	0,692	
	& interaction	0,440	0,194	5,695	0,001	0,085	1	54	0,772	0,894
right caudal anterior cingulate	& main effects	0,441	0,194	5,641	0,013	0,431	2	55	0,652	
	& interaction	0,444	0,197	5,681	0,003	0,222	1	54	0,639	0,894
right precuneus	& main effects	0,476	0,227	5,525	0,045	1,613	2	55	0,208	
	& interaction	0,617	0,381	4,989	0,154	13,467	1	54	0,001	0,016
right cuneus	& main effects	0,443	0,196	5,634	0,015	0,499	2	55	0,610	
	& interaction	0,497	0,247	5,502	0,051	3,668	1	54	0,061	0,353
right fusiform	& main effects	0,438	0,192	5,647	0,011	0,368	2	55	0,694	
	& interaction	0,440	0,193	5,695	0,001	0,082	1	54	0,776	0,894
right insula	& main effects	0,444	0,197	5,631	0,015	0,524	2	55	0,595	
	& interaction	0,456	0,208	5,642	0,012	0,785	1	54	0,379	0,759
right amygdala	& main effects	0,463	0,215	5,568	0,033	1,163	2	55	0,320	
	& interaction	0,464	0,215	5,618	0,000	0,033	1	54	0,856	0,894
right caudate	& main effects	0,439	0,193	5,646	0,011	0,379	2	55	0,686	
	& interaction	0,439	0,193	5,696	0,000	0,031	1	54	0,862	0,894
right putamen	& main effects	0,497	0,247	5,451	0,066	2,408	2	55	0,099	
	& interaction	0,501	0,251	5,487	0,004	0,283	1	54	0,597	0,880

Hierarchical model with 3 steps: 1st step including covariates of age and sex; 2nd step including main effects of group and Region of interest; 3rd step including interaction between main effects. Abbreviations: LEAS = Levels of emotional awareness scale, SE = standard error, df = degrees of freedom, FDR = false discovery rate.

Supplementary table 3. Gray matter volume partial correlations with Levels of emotional awareness scale.

	Healthy controls[a]				Schizophrenia[b]			
	LEAS self		LEAS other		LEAS self		LEAS other	
**Region of interest**	**rho**	**p**	**rho**	**p**	**rho**	**p**	**rho**	**p**
left lateral orbitofrontal	0,17	0,568	0,04	0,994	-0,60	0,147	-0,43	0,501
left medial orbitofrontal	-0,38	0,568	-0,31	0,686	-0,36	0,558	-0,40	0,501
left caudal middle frontal	-0,07	0,886	<0,01	0,994	-0,03	0,972	0,28	0,657
left rostral middle frontal	-0,05	0,886	-0,03	0,994	-0,16	0,807	-0,10	0,973
left superior frontal	-0,22	0,568	-0,10	0,994	-0,32	0,628	-0,04	0,973
left rostral anterior cingulate	0,30	0,568	0,36	0,642	0,27	0,628	0,20	0,917
left caudal anterior cingulate	-0,09	0,846	0,02	0,994	-0,03	0,972	-0,07	0,973
left precuneus	0,23	0,568	-0,01	0,994	-0,31	0,628	-0,35	0,506
left cuneus	-0,30	0,568	-0,06	0,994	0,36	0,558	0,03	0,973
left fusiform	0,22	0,568	0,17	0,994	-0,14	0,807	-0,45	0,501
left insula	0,23	0,568	0,45	0,336	-0,20	0,782	-0,29	0,651
left amygdala	0,25	0,568	-0,09	0,994	0,03	0,972	0,07	0,973
left caudate	0,45	0,384	0,25	0,994	-0,02	0,972	0,12	0,973
left putamen	0,18	0,568	-0,18	0,994	-0,04	0,972	-0,01	0,973
right lateral orbitofrontal	0,15	0,623	0,08	0,994	-0,39	0,558	-0,11	0,973
right medial orbitofrontal	<0,01	0,998	0,34	0,642	-0,15	0,807	-0,39	0,501
right caudal middle frontal	0,02	0,989	-0,02	0,994	-0,05	0,972	0,26	0,661
right rostral middle frontal	0,18	0,568	0,14	0,994	-0,17	0,807	-0,03	0,973
right superior frontal	-0,34	0,568	-0,01	0,994	-0,28	0,628	-0,04	0,973
right rostral anterior cingulate	0,19	0,568	0,07	0,994	-0,09	0,972	0,04	0,973
right caudal anterior cingulate	0,17	0,568	-0,08	0,994	0,02	0,972	0,06	0,973
right precuneus	0,05	0,886	0,04	0,994	-0,38	0,558	-0,68	0,028
right cuneus	-0,22	0,568	0,15	0,994	0,26	0,628	-0,05	0,973
right fusiform	0,01	0,989	0,14	0,994	0,37	0,558	-0,01	0,973
right insula	-0,05	0,886	0,09	0,994	-0,15	0,807	-0,08	0,973
right amygdala	0,09	0,846	-0,12	0,994	-0,21	0,782	-0,30	0,651
right caudate	0,27	0,568	0,04	0,994	-0,01	0,972	0,12	0,973
right putamen	0,20	0,568	-0,20	0,994	-0,28	0,628	-0,37	0,501

[a] with age, sex and MCCB (neurocognition) as covariates.
[b] with age, sex, MCCB (neurocognition), number of episodes, PANSS Total and medication (in chlorpromazine eqivalents) as covariates.
